# Static and Dymanic disorder in Formamidinium Lead Bromide Single Crystals

**DOI:** 10.1063/4.0000963

**Published:** 2025-10-27

**Authors:** Yael Diskin-Posner

**Affiliations:** Weizmann Institute, Rehovot, Rehovot, Israel

## Abstract

The superior and tunable optoelectronic properties of lead halide perovskite thin films have been used to improve the device performance of solar cells, photodetectors, and light-emitting diodes. The material properties of lead halide perovskite thin films play important roles in determining the performance of optoelectronic devices. It is important to correctly understand the relationship between the material properties and device performance in order to realize optimal results.

Lead halide perovskites with the general formula APbX_3_ [A=CH_3_NH_3_^+^, CH(NH_2_)_2_^+^ or Cs^+^; X =I^-^, Br^-^, or Cl^-^], have gained much attention as photovoltaic materials because of their high power conversion efficiency (PCE) of over 22%. We focused on the family of the lead bromide perovskites, and specifically on formamidinium lead Bromide crystals.

Our study combines a high resolution single-crystal X- ray diffraction with THz-range Raman-scattering and first-principles calculations to probe the inorganic sub-lattice dynamics evolution with temperature in the range of 100 - 300 K. The study shows that formamidinium lead bromide is unique, because of its inorganic sub-lattice exhibits intrinsic local static disorder, that co-exists with a well-defined average crystal structure.

